# Monitoring the Crosstalk Between the Estrogen Receptor and Human Epidermal Growth Factor Receptor 2 with PET

**DOI:** 10.1007/s11307-020-01496-7

**Published:** 2020-04-13

**Authors:** I. F. Antunes, G. A. P. Hospers, J. W. A. Sijbesma, A. S. Boerema, A. van Waarde, A. W. J. M Glaudemans, R. A. J. O. Dierckx, E. G. E. de Vries, E. F. J. de Vries

**Affiliations:** 1 Department of Nuclear Medicine and Molecular Imaging, University of Groningen, University Medical Center Groningen, Hanzeplein 1, 9713 GZ Groningen, The Netherlands; 2 Department of Medical Oncology, University of Groningen, University Medical Center Groningen, Hanzeplein 1, 9713 GZ Groningen, The Netherlands

**Keywords:** Positron emission tomography, Estrogen receptor, Human epidermal growth factor receptor, Crosstalk, [^18^F]FES, Imaging

## Abstract

**Purpose:**

Ovarian cancer (OC) leads to poor survival rates mainly due to late stage detection and innate or acquired resistance to chemotherapy. Thus, efforts have been made to exploit the estrogen receptor (ER) and human epidermal growth factor receptor 2 (HER2) to treat OC. However, patients eventually become resistant to these treatments as well. HER2 overexpression contributes to the acquired resistance to ER-targeted treatment. Trastuzumab treatment, on the other hand, can result in increased expression of ER, which, in turn, increases the sensitivity of the tumors towards anti-estrogen therapy. More insight into the crosstalk between ER and HER2 signaling could improve our knowledge about acquired resistance in ovarian cancer. The aim of this study was to evaluate whether PET could be used to detect changes in ER expression induced by HER2-targeted treatment *in vivo*.

**Procedures:**

Male athymic nude mice were subcutaneously (sc) inoculated with 10^6^ SKOV3 human ovarian cancer cells (HER2+/ER+). Two weeks after inoculation, tumor-bearing mice were treated intraperitoneally with either vehicle, the HER2 antibody trastuzumab (20 mg/kg, 2×/week), or the HER2-tyrosine kinase inhibitor lapatinib (40 mg/kg, 5 days/week) for 2 weeks. Thereafter, ER expression in the tumor was assessed by PET imaging with 16α-[^18^F]-fluoro-17β-estradiol ([^18^F]FES). Tumors were excised for *ex vivo* ER and HER2 measurement with Western blotting and immunohistochemistry.

**Results:**

All treatments led to smaller tumors than vehicle-treated tumors. Higher [^18^F]FES maximum standardize tumor uptake (SUV_max_) was observed in animals treated with trastuzumab (+ 29 %, *P* = 0.002) or lapatinib (+ 20 %, *P* = 0.096) than in vehicle-treated controls. PET results were in agreement with *ex vivo* analyses.

**Conclusion:**

FES-PET imaging can detect changes in ER expression induced by HER2-targeted treatment and therefore can be used to investigate the crosstalk between ER and HER2 in a noninvasive manner.

## Introduction

Ovarian cancer is often detected at a late stage, leading to poor survival rates. Despite a favorable initial response to chemotherapy, epithelial ovarian cancer (EOC) remains with a high rate of recurrence. Efforts have been made to exploit the estrogen dependency of ovarian cancer cells to treat EOC with endocrine therapies. In contrast to other hormonal-dependent cancers, such as breast cancer, anti-estrogen therapies only proved to be successful in a small number of ovarian cancer (OC) patients, and eventually, most patients acquire resistance towards endocrine treatment [1, 2]. The molecular mechanisms contributing to the development of this spontaneous or acquired resistance are not yet completely elucidated. Increasing evidence suggests that aberrant activation of alternative signaling cascades, such as growth factor-mediated signaling, can provide the proliferation and survival signals to circumvent the effects of endocrine treatment [3]. Expression of the human epidermal growth factor receptor 2 (HER2) in ovarian cancer has been associated with an advanced stage of the disease, a higher recurrence rate, and shorter survival rate [4, 5].

HER2-targeted therapies, including the antibody trastuzumab and the tyrosine kinase inhibitor lapatinib, have shown some efficacy towards certain HER2-overexpressing ovarian cancers, but eventually acquired resistance to these therapies was also observed [6]. Trastuzumab treatment can increase the expression of ER by tumor cells, which, in turn, increases the sensitivity of the tumors towards anti-estrogen therapy [7]. These results suggest that there is a treatment-induced crosstalk between estrogen receptors (ER) and HER-2 signaling pathways. Thus, a better understanding of this crosstalk might contribute to optimal treatment selection.

To obtain more insight into the ER-HER2 crosstalk, a quantitative assessment of the expression levels of these receptors in tumor lesions can be very useful. Currently, ER and HER2 expression are usually determined *ex vivo* in the primary tumor or a biopsy of a metastasis. However, the receptor status of metastases may differ from that in the primary tumor and can differ across metastases within a single patient [8]. In addition, receptor expression in the tumor lesions can change over time, either spontaneously or due to treatment. Thus, biopsy results only capture a part of the picture. In contrast to a biopsy, functional imaging techniques, such as positron emission tomography (PET) with 16α-[^18^F]-fluoro-17β-estradiol ([^18^F]FES) as the tracer, can noninvasively visualize the ER expression across all tumor lesions throughout the body, respectively. Thus, PET might be used to monitor the receptor status of all tumor lesions in one patient simultaneously. The aim of this preclinical study was, therefore, to evaluate whether [^18^F]FES-PET could be used for the *in vivo* detection of changes in ER expression induced by HER2-targeted treatment.

## Materials and Methods

### Cell Lines and Animals

The human ovarian cancer cell line SKOV3 (American Type Culture Collection) was grown in Dulbecco’s Modified Eagle Medium (DMEM)-high glucose cell culture medium, supplemented with 10 % fetal calf serum. Cells were cultured at 37 °C in a humidified atmosphere of 95 % air and 5 % CO_2_ (v/v). SKOV3 cells express both ER and HER2, but are not dependent on estrogens for survival and proliferation. Thus in this study, non-castrated athymic nude male mice (Hsd, Athymic nude-*Foxn1*^*nu*^, 6–8 weeks old, *n* = 31) were used to exclude any effect of the highly variable circulating estrogen levels during the menstrual cycle in females on PET tracer binding. We decided not to use ovariectomized female mice, since this is a proof-of-concept study and wanted to minimize animal discomfort. Since the estrogen levels are much lower in male than in female mice and consequently have negligible impact on PET imaging results, we decided not to castrate the male mice [9]. Mice were obtained from Envigo (formerly Harlan, Lelystad, The Netherlands), and upon arrival, they were provided with standard laboratory chow and tap water *ad libitum*. All studies were carried out in compliance with the Dutch regulations for animal experiments. The protocol was approved by the Institutional Animal Care and Use Committee of the University Medical Center Groningen (protocol number: DEC 6657C). After at least 1 week of acclimatization, SKOV3 cells (1 × 10^6^ cells in a 1:1 mixture of Matrigel and DMEM-high with 10 % fetal calf serum) were subcutaneously (sc) injected into the upper back of the mice. Two weeks after inoculation, palpable tumor nodules had formed in most of the animals. Due to the ulceration of the tumors, 5 animals had to be sacrificed before the beginning of treatment.

### Tumor Volumes and Therapeutic Regimens

All treatments were started 2 weeks after tumor inoculation. SKOV3 tumor-bearing mice were randomly assigned to a treatment group: (1) trastuzumab (*n* = 8, 20 mg/kg intraperitoneally (ip)) [7] or vehicle (*n* = 6, saline) twice a week or (2) lapatinib (*n* = 6, 40 mg/kg ip) [10] or vehicle (*n* = 6, saline) 5 days/week. Tumor diameters were measured with a caliper, and tumor volume was calculated using the following formula: [length × (width)^2^]/2. Relative tumor volumes were calculated for each individual tumor by dividing the tumor volume at the end of the treatment by the tumor volume at the beginning of the treatment.

### PET and CT Imaging

[^18^F]FES was produced as previously described [11]. Imaging was conducted using a micro-PET Focus 220 rodent scanner (CTI Siemens). In mice that were treated with either trastuzumab (ip, *n* = 8), lapatinib (ip, *n* = 6), or saline (ip, *n* = 6), [^18^F]FES (13.7 ± 1.9 MBq) was administered *via* penile vein injection (100 μl). Thirty minutes post-injection, a static emission scan was acquired for 30 min. After completion of the PET scan, the animals were sacrificed with an overdose of anesthesia, and a 15-min transmission scan with a Co-57 point source was acquired for the correction of scatter and attenuation of 511 keV photons by tissue. After the transmission scan, the animal remained fixed to the bed, and the bed was positioned in a CT scanner (MicroCAT II, CTI Siemens). A 15-min CT scan was acquired for anatomic localization of the tumor (exposure time = 1050 ms; X-ray voltage = 55 kVp; anode current = 500 μA; number of rotation steps = 500; total rotation = 360 °).

After completion of the imaging procedures, the tumors were excised and weighted. Tumor samples were either formalin-fixed paraffin embedded for immunohistochemical analyses or snap frozen in liquid nitrogen and stored at − 80 °C for *ex vivo* Western blotting assays.

### PET Data Analysis

Emission sinograms were iteratively reconstructed (OSEM2D) after being normalized and corrected for scattering, attenuation, and decay of radioactivity. The PET and CT images were fused using Inveon Research Workplace software (Siemens Preclinical Solution). For data analysis, a volume of interest (VOI) was manually drawn around the whole tumor on the CT image and transferred to the corresponding PET image. A second VOI of the viable part of the tumor was generated automatically using a region growing method with a threshold of 50 % of the hottest pixel in the tumor. The resulting VOIs were used to generate the corresponding tracer accumulation in the viable part of the tumor, expressed as kBq/cc, using Inveon Research Workplace software. The tracer concentration was converted into a maximum standardized uptake value (SUV_max_), which was defined as follows: [maximum tissue activity concentration (Bq/cc) × body weight (g)/injected dose (Bq)] and assuming a tissue density of 1 g/cc. To correct for non-specific binding, correction for background was applied, using a VOI in the contralateral shoulder muscle as background. The background-corrected SUV was calculated as follows: SUV_corr_ = SUV_tumor_–SUV_muscle_.

### Western Blotting

On average, 43 ± 15 mg tumor tissue was homogenized in ice-cold commercial radio-immune precipitation assay (RIPA) buffer (Pierce, lot# 89901) supplemented with Complete-Mini Protease Inhibitor Tablets (Roche lot# 10810400). RIPA buffer was added in a 1:15 tissue-to-buffer ratio. Samples were first ground using a motorized tissue grinder, followed by sonication for 2 × 5 s. Samples were vortexed and incubated for 45 min on a shaker and subsequently centrifuged at 20841*g* for 20 min at 4 °C. The clear supernatant was transferred to new tubes. The protein concentrations were determined by Bradford assay and standardized to 2.0 mg/ml by adding ice-cold RIPA buffer. Samples were subsequently aliquoted and stored at − 80 °C until further processing.

The frozen samples were thawed and mixed 4× with lithium dodecyl sulfate (LDS) sample buffer (Life Technologies) and dithiothreitol (DTT) as a reducing agent (Life technologies) in the following ratio: sample:LDS:DTT = 0.65:0.25:0.10. Samples were mixed and heated for 10 min at 70 °C. Samples were loaded and proteins were separated on Novex® Bolt Bis-Tris 4–12 % gradient gels (Life technologies, lot# 15031091) using 30 μg of total protein per well utilizing the 3-(N-morpholino)propanesulfonic acid (MOPS) buffer system. Proteins were transferred from gel to a 0.2-μm polyvinylidene difluoride membrane (Life technologies), using the I-blot dry blotting system (Life Technologies).

Membranes were blocked with 0.2 % I-block (Tropix) and probed with rabbit-anti-C-erbβ-2, 1:500 (Thermo Scientific; ref# RM_9103_S; lot#910351202E), mouse-anti-ER-α (F10) (Santa Cruz; ref# sc-8002; lot# G0908), and mouse-anti-β-actin 1:1000000 (MP-Biomedicals; ref# 08691002), followed by the secondary antibodies donkey-anti-rabbit-horseradish peroxidase (HRP), 1:5000 (GE-Healthcare; ref# NA934V), or goat anti-mouse-HRP (Santa- Cruz; ref# SC-2005). Detection was done using standard enhanced chemoluminescence (Thermo Fisher) in a Chemidoc-XRS+ imaging system (Biorad). Quantification was performed in the ImageLab 5.0 (Biorad). The protein of interest was expressed as a fraction of the β-actin signal.

### Immunohistochemical Analysis

Immunohistochemical analysis of the tumors treated with trastuzumab (*n* = 2) or vehicle (*n* = 3) was performed to confirm the changes in ER expression observed by [^18^F]FES -PET. The immunohistochemical staining of the formalin-fixed paraffin-embedded SKOV3 tumors was performed according to the routine protocol for clinical samples. ER-α rabbit monoclonal primary antibody (Clone SP-1, Ventana) and a *c–erb*B-2/HER-2/*neu* rabbit monoclonal antibody (Clone SP-3, Thermo Fisher scientific) were used for immunohistochemical staining of ER-α and HER2, respectively. HER2 and ER immunohistochemistry results were analyzed by an expert histopathologist according to a 4-level scoring system: 0, 1, 2, and 3+ for no, weak, moderate, and high-intensity staining, respectively.

### Statistical Analysis

Statistical analyses were performed with Excel 2003 (Microsoft) and GraphPad Prism (version 5.04). Differences in tracer accumulation between vehicle controls and treated animals were analyzed using a two-sided unpaired Student’s *t* test. Significance was reached when the probability (P) was < 0.05. Throughout the manuscript, values are presented as mean ± the standard deviation, unless it is stated otherwise.

## Results

### Tumor Growth and Body Weight

**Fig. 1:**
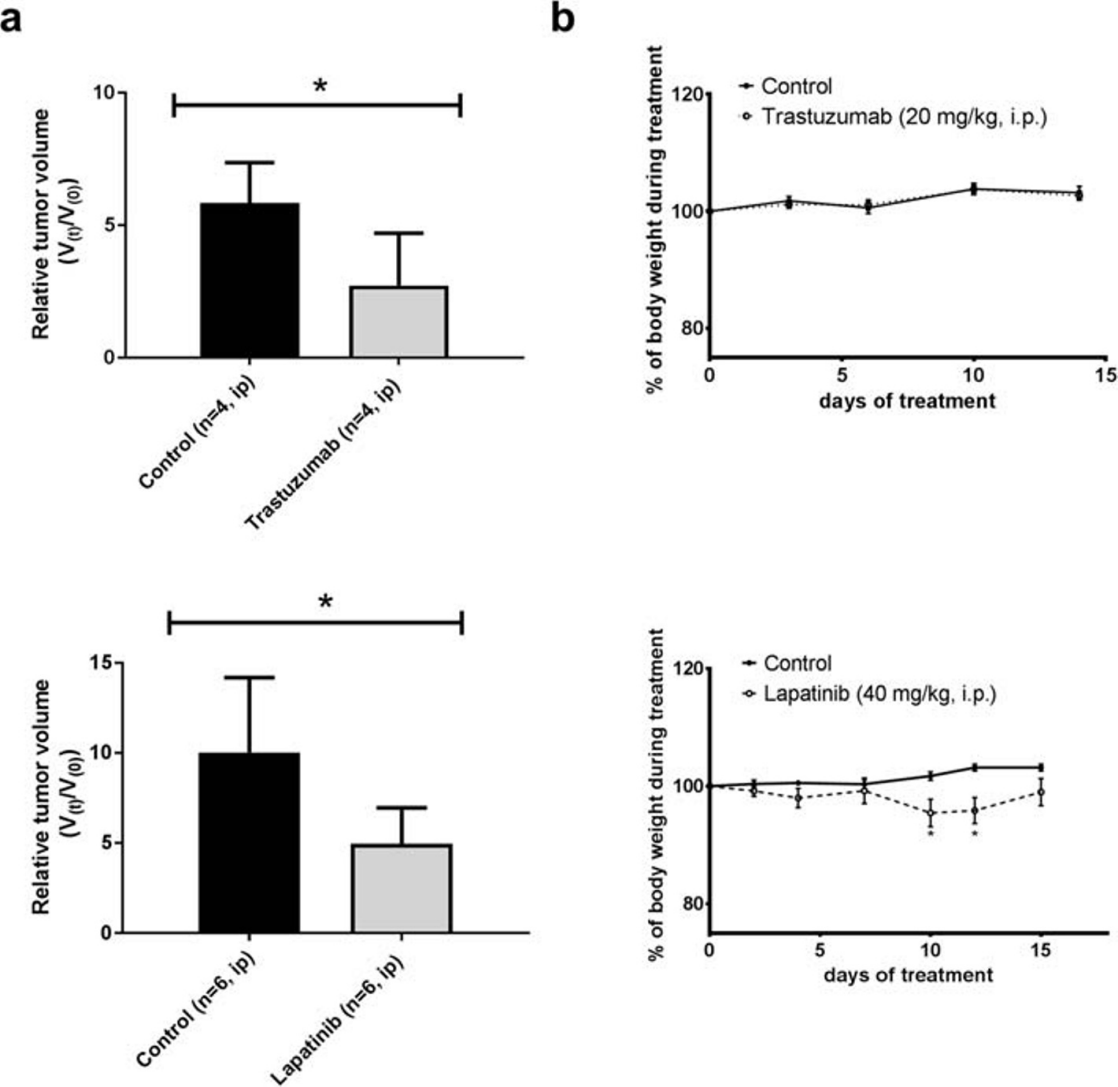
**a** Relative tumor volume in nude mice where V(0) is the tumor volume before treatment and V(t) is the tumor volume at the end of treatment. Six animals are unaccounted in this graph since their xenografts were not measurable at the beginning of the treatment. **b** % Body weight of mice with a subcutaneous SKOV-3 xenograft during treatment with targeted anticancer drugs (dashed line) and corresponding vehicle controls (solid line), considering the weight at the start of treatment as 100 %. Results are expressed as mean ± SEM. Statistically significant differences compared with controls are indicated with *P < 0.05.

The tumor size at the start of treatment did not differ significantly between treatment (0.22 ± 0.15 cm^3^) and control groups (0.16 ± 0.09 cm^3^, *P* = 0.59). All treatments delayed tumor growth compared with the corresponding control groups (Fig. 1a). None of the treatments caused any severe side effects. Mice treated with lapatinib had a significantly lower body weight than control animals on day 10 and 12 of treatment, but at the time of the PET scan (day 15), there was no significant difference in body weight between the treatment and control group anymore (Fig. 1b).

### Pet

**Fig. 2:**
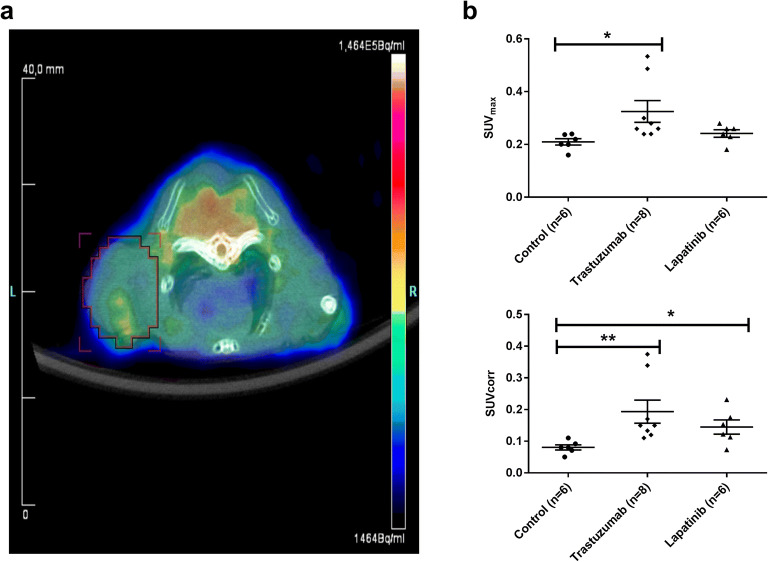
**a** Representative [^18^F]FES-PET-CT fusion image of a mouse bearing a SKOV3 xenograft treated with trastuzumab. The tumor is delineated by the red region of interest. Quantitative ^18^F-FES uptake (**b**) as SUV_max_ and (**c**) as SUV_corr_ in SKOV3 xenografts of mice treated with trastuzumab, lapatinib, or vehicle (*n* represents the number of animals). Statistically significant differences *P* < 0.05 or *P* < 0.001 compared with controls are indicated with * or **, respectively.

[^18^F]FES uptake in SKOV3 xenografts of mice treated with trastuzumab (SUV_max_ 0.27 ± 0.02; *P* = 0.002) was approximately 29 % higher than that in controls (Fig. 2a, b). [^18^F]FES uptake in the SKOV3 xenografts of lapatinib-treated mice (SUV_max_ 0.25 ± 0.05) was also increased by 20 % compared with vehicle-treated controls (SUV_max_ 0.21 ± 0.04), but this difference did not reach statistical significance (*P* = 0.096). There was no significant difference between animals treated with trastuzumab or lapatinib (*P* = 0.385). After correction of the tracer uptake for background, the effects of treatment became even more pronounced. The SUV_corr_ of [^18^F]FES (Fig. 2c) was significantly higher in both the trastuzumab-treated (0.14 ± 0.02, *P* = 0.0002) and the lapatinib-treated SKOV3 xenografts (0.14 ± 0.06, *P* = 0.024) than in non-treated controls (0.08 ± 0.03).

### Western Blotting

**Fig. 3:**
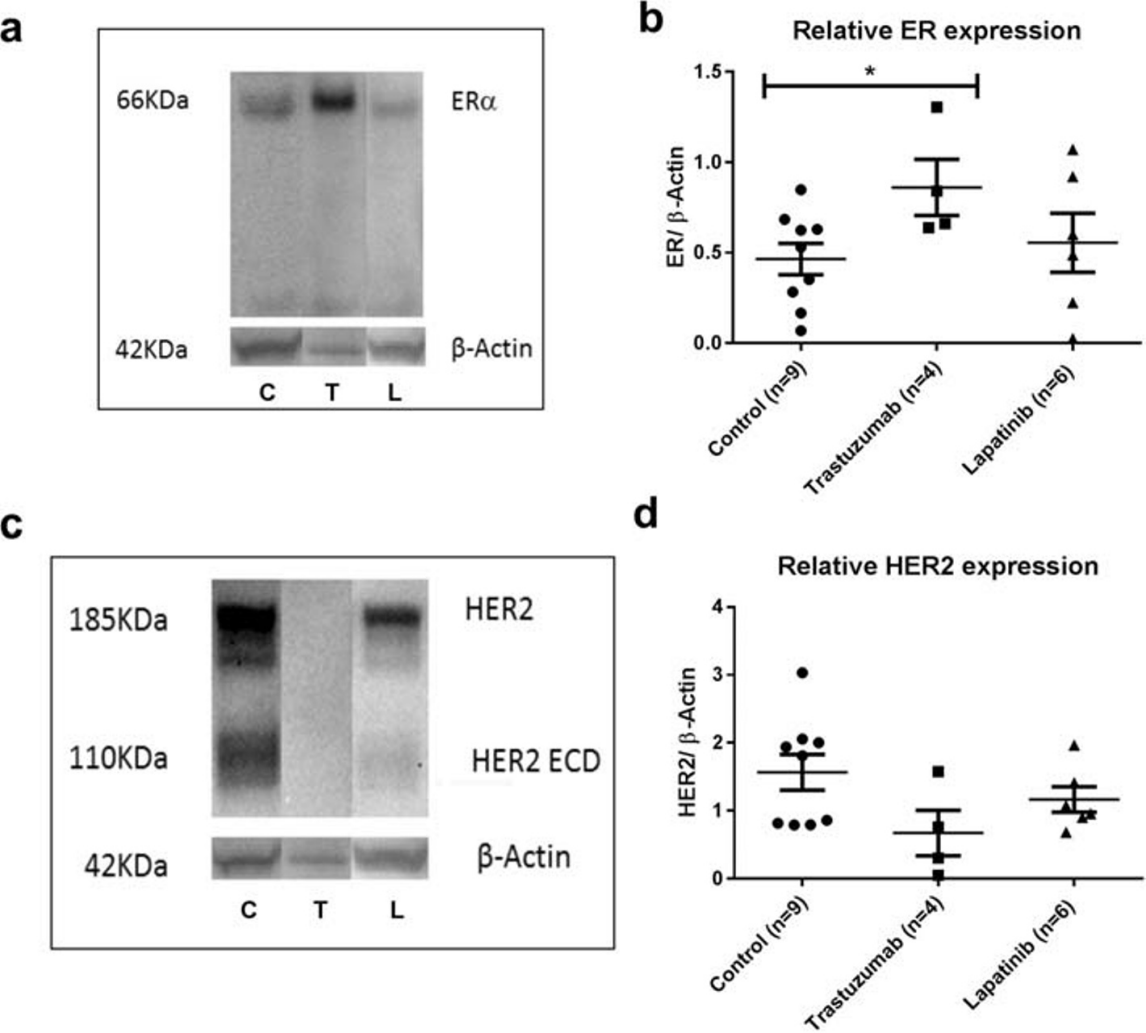
A representative example of the Western blot analysis of ERα (**a**) or HER2 (**c**) in SKOV-3 tumor xenografts from mice treated with trastuzumab (T), lapatinib (L), or vehicle (C). Western blot analysis of (**b**) ERα to β-actin protein ratio and (**d**) full-length HER2 to β-actin protein ratio in SKOV-3 tumor xenografts (*n* represents the number of mice). Statistically significant differences *P* < 0.05 compared with controls are indicated with *.

Western blotting of the SKOV3 xenografts showed an immunoreactive band for ERα at 66 kDa (Fig. 3a). For HER2, immunoreactive bands were observed at 110 kDa for the extracellular domain (HER2 ECD) and at 185 kDa for the total receptor (HER2) (Fig. 3c). The ERα/β-actin ratio found in trastuzumab-treated mice (0.86 ± 0.27) was 1.8 times higher than in controls (0.47 ± 0.25, *P* = 0.036) while the ERα/β-actin ratio in lapatinib-treated mice (0.56 ± 0.36, *P* = 0.604) was about 20 % higher than in controls, although this effect did not reach statistical significance (Fig. 3b).

The HER2 expression levels in tumors obtained from mice treated with trastuzumab (0.67 ± 0.58) were 2.3 times lower that in controls (1.57 ± 0.75, *P* = 0.077) while the HER2/β-actin ratio in lapatinib-treated mice (1.16 ± 0.42, *P* = 0.288) was only 1.3 times lower than in controls (Fig. 3d).

### Histology and Immunohistochemistry

**Fig. 4:**
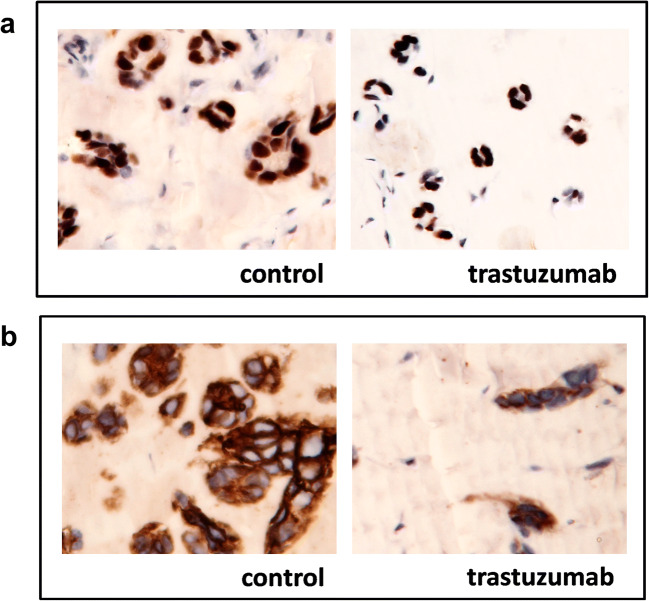
Representative example of the immunohistochemical analysis of ERα (**a**) or HER2 (**b**) expression in SKOV-3 tumor xenografts from vehicle-treated control mice and mice treated with trastuzumab, × 20 magnification.

The increase in ER expression after trastuzumab treatment observed by PET imaging and Western blotting was confirmed by immunohistochemistry. Tumor tissue following trastuzumab treatment showed less viable tumor cells. In the remaining viable cells, the HER2 staining (Fig. 4b) was found to be less intense (score + 2) than in untreated tumors (score + 3). However, ERα staining in the remaining viable SKOV3 (Fig. 4a) tumor cells was more intense (score + 2) than the staining of untreated SKOV3 tumors (score + 1).

## Discussion

This study shows that [^18^F]FES-PET can detect changes of ER expression in a preclinical xenograft model as a result of inhibition of the HER2 signaling pathway with trastuzumab. These results were supported by immunohistochemistry and Western blotting. This is the first study in which crosstalk between the HER2 and ER signaling pathways induced by treatment was visualized and quantified noninvasively with PET.

Trastuzumab is a monoclonal antibody that binds to HER2 and induces internalization and downregulation of the receptor. In this study, we observed that trastuzumab treatment can also affect the ER signaling pathway. [^18^F]FES-PET showed approximately 29 % higher tracer uptake (SUV_max_) in SKOV3 xenografts of mice treated with trastuzumab when compared with control mice. When background correction was taken into account (SUV_corr_), the effect of treatment on specific tracer uptake was even more pronounced (70 %), indicating a trastuzumab treatment-induced increase in ER expression. In this study, we did not perform full pharmacokinetic modeling of ^18^F-FES uptake but derived the SUV from a static scan. Theoretically, the SUV is sensitive to changes in several physiological parameters, and the effect of trastuzumab treatment on the [^18^F]FES uptake may, therefore, be affected by a trastuzumab-induced increase in tumor perfusion (tracer delivery) or decrease in tracer metabolism. In addition, the *ex vivo* analysis of the tumors by Western blotting and immunohistochemical staining confirmed the treatment-induced increase in ERα expression.

Lapatinib can inhibit downstream HER2 signaling by antagonizing the kinase activity of HER2, leading to inhibition of cell proliferation and apoptosis induction and antitumor activity in patients [12, 13]. However, part of the patients is resistance to lapatinib treatment, and among the ones that do respond to the treatment, the disease will eventually progress. Wang et al. found that lapatinib may induce the activation of an alternative pathway, such as ER-mediated signaling which could cause resistance to lapatinib treatment in HER2-positive breast cancer cells [14]. Therefore, we investigated if lapatinib treatment-induced changes in ER receptor expression could be detected by PET with the tracer [^18^F]FES. In this study, 14-day lapatinib treatment resulted in 54 % growth reduction of the SKOV3 xenografts and a 20 % increase in [^18^F]FES uptake (SUV_max_) in SKOV3 xenografts, although these effects were not statistically significant (*P* = 0.09). The lack of significance could be due to the high inter-subject variability. However, it seems more likely that lapatinib had an effect on the non-specific uptake of the tracer. In fact, we observed that background [^18^F]FES uptake in the contralateral muscle was significantly lower in the lapatinib-treated group than in controls, which suggests that lapatinib could have affected [^18^F]FES clearance from circulation (data not shown). When [^18^F]FES uptake was corrected for background, lapatinib-treated xenografts showed significantly higher specific tracer uptake (SUV_corr_) than in non-treated xenografts. Lapatinib-induced changes in [^18^F]FES uptake appear to be in agreement with our *ex vivo* analyses, as Western blotting also indicated a high variability between tumors and a (statistically not significant) 20 % increase in ER expression.

In our preclinical study, [^18^F]FES uptake in SKOV3 xenografts (SUV_max_ 0.21–0.27) was relatively low when compared with tracer uptake in the breast or ovarian cancer in patients (SUV 1.5–6.0) [15, 16]. Such low [^18^F]FES uptake was also observed in other preclinical studies in tumor-bearing mice [17, 18]. A plausible cause for the low uptake of [^18^F]FES in this study is the relatively low expression of ER in the SKOV3 tumor cell line (*ca.* 830 fmol/mg of protein) compared with, for example, the frequently used ER-expressing MCF7 breast tumor cell line (*ca.* 1900 fmol/mg of protein) [18, 19].

A limitation of our study was the relatively small number of animals per treatment, especially taking the high inter-subject variability in tumor growth and treatment response into account. Repetitive imaging could help to overcome this hurdle, as it would enable the assessment of the changes in receptor expression within a single animal over time. This would increase the statistical power of the study, as the data could be analyzed by paired tests.

Another limitation is that we only tested the SKOV3 xenograft model in a male mice model to show proof-of-concept. Ideally, our findings should be confirmed in a more relevant model, such as a xenograft model in ovariectomized female mice, preferably using tumor cells that express higher amounts of both ER and HER2 (HER2+/ER+), such as BT474 breast cancer cells. However, we have now shown proof-of-concept, and considering that PET is a highly translational technique, confirmation of our findings can also be obtained directly in cancer patients. [^18^F]FES-PET was already shown to be able to detect ER expression in the tumors of patients with epithelial ovarian cancer and thus may also be used for the detection of treatment-induced crosstalk between HER2 and ER in cancer patients.

A potential concern for assessing ER expression in ovarian cancer patients could be the location of the tumor in the abdominal area where visualization can be hampered by the high physiological background uptake of [^18^F]FES in the liver, gallbladder, intestines, ovaries, and bladder [20]. Despite this limitation, [^18^F]-FES-PET/CT was shown to have a high sensitivity and specificity (79 % and 100 %) in patients with epithelial ovarian cancer, comparable with its performance in patients with metastasized breast cancer (sensitivity 84 %; specificity 98 %) [20]. [^18^F]FES-PET can thus be expected to be sufficiently sensitive and specific to study ER-HER2 crosstalk in patients with ovarian cancer, especially in solid tumors larger than 10 mm. For diffuse tumors and solid tumors with smaller dimensions, this will remain challenging. Needless to say, [^18^F]FES-PET could also be used to evaluate treatment-induced crosstalk between the ER and HER2 signaling pathways in breast cancer patients.

## Conclusion

This proof-of-concept study shows that PET imaging with [^18^F]FES could be used to visualize and quantify the treatment-induced crosstalk between the ER and HER2 signaling pathways in a preclinical cancer model. Since the same PET method can be used in humans, this technique can be applied to translate preclinical findings on the crosstalk between signaling pathways into a clinical setting.
